# Non-Deterministic Semantics for Quantum States

**DOI:** 10.3390/e22020156

**Published:** 2020-01-28

**Authors:** Juan Pablo Jorge, Federico Holik

**Affiliations:** 1 Physics Department, University of Buenos Aires, Buenos Aires 1428, Argentina; jorgejpablo@gmail.com; 2 Instituto de Física La Plata, UNLP, CONICET, Facultad de Ciencias Exactas, 1900 La Plata, Argentina

**Keywords:** quantum states, non-deterministic semantics, truth functionality

## Abstract

In this work, we discuss the failure of the principle of truth functionality in the quantum formalism. By exploiting this failure, we import the formalism of N-matrix theory and non-deterministic semantics to the foundations of quantum mechanics. This is done by describing quantum states as particular valuations associated with infinite non-deterministic truth tables. This allows us to introduce a natural interpretation of quantum states in terms of a non-deterministic semantics. We also provide a similar construction for arbitrary probabilistic theories based in orthomodular lattices, allowing to study post-quantum models using logical techniques.

## 1. Introduction

According to the principle of truth-functionality or composability (TFP), the truth-value of a complex formula is uniquely determined by the truth-values of its subformulas. It is a basic principle of logic. However, as explained in [1], many real-world situations involve dealing with information that is incomplete, vague or uncertain. This is especially true for quantum phenomena, where only probabilistic assertions about possible events can be tested in the lab. These situations pose a threat to the application of logical systems obeying the principle of truth-functionality in those problems. As is well known, the TFP fails in many logical systems of interest (see examples in [2]).

One possible way to deal with this situation is to relax the TFP. This is the path followed in [3], where the idea of non-deterministic computations—borrowed from automata and computability theory—is applied to evaluations of truth-values of formulas. This leads to the introduction of non-deterministic matrices (N-matrices). These are a natural generalization of ordinary multi-valued matrices, in which the truth-value of a complex formula can be chosen non-deterministically, out of some non-empty set of options [1,3,4,5].

The Kochen-Specker theorem [6] is one of the most fundamental no-go theorems of quantum mechanics, and it holds in many probabilistic models of interest [7,8,9]. It has far-reaching consequences for the interpretation of the quantum formalism (see for example [10]). In particular, it imposes very strong restrictions on the possible physically motivated valuations that can be defined over the propositions associated with quantum models. In this work, we discuss in which sense the principle of truth functionality is false in the quantum formalism and describe how N-matrices can be used to describe quantum states and quantum state spaces. This novel perspective opens the door to new fundamental questions by introducing the possibility of interpreting quantum probabilities as a particular class of non-classical valuations.

We also extend our approach to a family of generalized probabilistic models—including quantum and classical probabilistic ones as particular cases. The N-matrices associated with classical probabilistic models are non-deterministic, but they always admit global classical valuations to the set {0,1}. These global valuations will not exist, in general, for non-classical probabilistic models. The study of post-quantum theories is a very active field of research nowadays, since it provides an extraordinary ground for studying the fundamental principles that underly the quantum formalism (see, for example, [11,12,13]). Furthermore, the study of contextual systems outside the quantum domain [14,15,16] poses the question of looking for contextual models which are non-quantum, but non-classical either. Furthermore, non-standard probabilities have been used to describe the deviations of classical logic in decision-making problems. As an example, negative probabilities have been applied to the study of inconsistent judgments [17]. Our extension could be useful for studying contextuality [18,19,20,21,22] in quantum mechanics and generalized probabilistic models from a novel logical perspective.

The logic-operational approach to quantum theory dates back to the 1930s, after the contribution of Birkhoff and von Neumann [23] (for further developments of the quantum logical approach, see [24,25,26,27,28,29,30,31,32,33,34,35,36,37,38]). More recently, a growing interest is put in describing logical structures associated to quantum computation [39,40,41,42].

In this work, we give an interesting turn to the quantum logical approach by introducing a relatively recently discovered logical technique into the foundations of quantum mechanics. As a result of our construction, we show that a quantum logical entailment arises by appealing to the quantum non-deterministic semantics. This is a step forward in the discussion whether there exists a well-behaved logic associated with the quantum formalism.

The paper is organized as follows. We start by reviewing the principle of truth functionality in classical logics in Section 2. Then, in Section 3, we discuss the Born rule and the Kochen-Specker and Gleason’s theorems from a quantum logical perspective. There, we discuss in which sense the principle of truth functionality is not valid in quantum mechanics. In Section 4, we review the formalism of N-matrix theory and non-deterministic semantics. In Section 5 we show how this formalism can be used to describe quantum states, as a particular set of valuations associated with infinite truth tables, that we first introduced here. We also discuss extensions to generalized probabilistic models and the detection of probabilities with finite precision. In Section 6 we discuss the possibility of developing a logical consequence in quantum logic. Finally, in Section 7, we draw our conclusions.

## 2. The Principle of Truth Functionality and Algebra Homomorphisms

In this section, we review the principle of truth functionality, in connection with the notion of classical semantics and algebra homomorphisms. The reader with a background in algebra and logic, may skip this section. We focus on the homomorphisms between a given algebra and the two-element Boolean algebra $B_{2}=\left\{0,1\right\}$, endowed with the usual operations (that we denote by $\tilde{\vee }$, $\tilde{\wedge }$, and $\tilde{\neg }$). The principle of truth functionality is very important for our work, because the KS theorem is expressed in terms of classical valuations satisfying a functional behavior with respect to truth-value assignments. We also discuss, in the following, how non-deterministic semantics can be used to deal with the failure of the TFP, and how they offer the possibility of understanding the consequences of the KS theorem under a new light.

In this section, we follow the treatment given in references [43,44]. Let us start with a definition of algebra that is relevant to our logical approach:

**Definition** **1.** *A type of algebras is a set F of function symbols, where each symbol f∈F has associated a natural number n, its arity.*

**Definition** **2.** *Given a type F of algebras, an algebra of type F is a pair A=〈A,F〉, where A is a set and $F={\left(f_{i}^{A}\right)}_{i\in I}$ is a family of operations over A, defined in such a way that, to each symbol of F of arity n, it corresponds an n-ary operation $f_{i}^{A}$. The set A is the universe associated with the algebra. Usually, one speaks about the algebra by referring to its universe only, in case that the operations are clearly understood from the context. We also write $f_{i}$ instead of $f_{i}^{A}$, where no confusion can arise.*

As an example, consider the algebra $B_{2}$. Its universe is the set {0,1} and its operations are given by $\tilde{\vee }$, $\tilde{\wedge }$ and $\tilde{\neg }$, with arity 2, 2 and 1, respectively.

### 2.1. Homomorphisms

A homomorphism between algebras is defined as follows:

**Definition** **3.** *Let A=〈A,F〉 and B=〈B,G〉 be algebras of the same type and let h:A→B be a function. The map h is a homomorphism from a A to B, if for any of n-ary symbol $f^{A}\in F$ we have that, for every n-tuple $\left(a_{1},...,a_{n}\right)$ of elements in A:*

$$h\left(f_{\left(a_{1},\ldots ,a_{n}\right)}^{A}\right)=f_{\left(h\left(a_{1}\right),...,h\left(a_{n}\right)\right)}^{B}$$

*being $f^{B}$ the operation in B which corresponds to $f^{A}$ in A.*

If a homomorphism *h* is a bijection, it is called an isomorphism.

Each propositional language and its well-formed formulas are defined by the set of connectives, whenever there is a denumerable set of propositional variables and punctuation symbols (under the assumption that we have recursive rules for their formation). Thus, a type is assigned to each language, in the same way as it occurs for algebras. For more discussion about this, we refer the reader to [45]. In order to illustrate these ideas, let us consider the implicational propositional calculus. The only connective of this calculus is “→”, which is binary. Thus, each formula which is not a variable is of the form a→b. This is a type 〈2〉 language. The classical propositional calculus with its connectives “¬”, “∨”, “∧” and “→”, is of the type 〈1,2,2,2〉.

We can evaluate the formulas of a language *L* in algebras of the same type, proceeding similarly as with homomorphisms between algebras. Valuations are defined in such a way that each *n*-ary connective is transformed into a corresponding *n*-ary operation. Valuations assign to each formula of the language an element of an algebra, that we may think of as its truth-value. In the classical case, this algebra is $B_{2}$, and 0 and 1 are identified as the values “true” and “false”, respectively. In the classical propositional calculus, this construction gives place to the well-known truth tables.

At this point, some readers might be interested in understanding with more detail the link between the type of a language and a type of algebra. For self completeness, we have included Section 2.1.1 below, in which we explain these notions with more detail.

We now give the definition of valuation for a propositional language:

**Definition** **4.** *Let L be a propositional language whose set of connectives is ${\left(c_{i}\right)}_{i\in I}$ and let 〈A,G〉 be an algebra where $G={\left(g_{i}\right)}_{i\in I}$. A (deterministic) valuation is a function v:L→A, such that for each n-ary connective c and formulas $B_{1},...B_{n}$, it satisfies*

$$v\left(c\left(B_{1},...,B_{n}\right)\right)=g^{c}\left(v\left(B_{1}\right),...,v\left(B_{n}\right)\right),$$

*being $g^{c}$ the n-ary operation in A corresponding to c.*

From the above definition of classical valuation, it follows that the value of $g^{c}\left(v\left(B_{1}\right),...,v\left(B_{n}\right)\right)$ is determined by the values $v(B_{1})$, … ,$v(B_{n})$, that the valuation *v* assigns to the propositions $B_{1}$, …, $B_{n}$ out of which the formula $c(B_{1},...,B_{n})$ is composed. This is the exact content of the principle of truth functionality. A truth functional operator is an operator whose values are determined by those of its components. All classical operators are truth-functional. Accordingly, classical propositional logic is a truth-functional propositional logic.

In classical propositional logic the algebra is given by $B_{2}$. Thus, for example, the value v(P∨Q) assigned by a classical valuation *v* to the compound proposition P∨Q is determined solely by the values v(P) and v(Q). In other words, we have $v\left(P\vee Q\right)=v\left(P\right)\tilde{\vee }v\left(Q\right)$. Similar examples can be given for $\tilde{\wedge }$ and $\tilde{\neg }$. In Section 3 we see how these notions can be extended to the quantum formalism.

#### 2.1.1. Types of Languages and Homomorphisms between Structures

For a detailed treatment of the content of this section, see [45]. Let us start with the definition of type:

**Definition** **5.** *A type (or signature) is a set τ of symbols which has the form:*

$$\tau =\left(\underset{1\leq n}{⋃}R_{n}\right)\cup \left(\underset{1\leq m}{⋃}F_{m}\right)\cup C,$$

*where $R_{n}$ is a set of relational n-arity symbols, $F_{m}$ is a set of m-arity functional symbols and C is a set of symbols for constants (or any other distinguished symbols of the system), which are referred as 0-arity functions.*

The relational and functional symbols acquire meaning only when they are considered in connection with a semantics or an interpretation. Thus, they are interpreted as relations, functions and distinguished elements, respectively, in a given universe of interpretation. It is necessary to define a language of a given type in order to apply the definitions of valuation and homomorphism, and then, to relate the notion of language with that of algebra (or structure in the more general case).

In this work, we will restrict ourselves to the propositional calculus only. We include here the definition of first-order languages, because it can be useful for the development of quantum-inspired languages.

**Definition** **6.** *The symbols for building expressions of a language of type τ—that we denote by $L_{\tau }$—are the following:*

*1*. *Individual variables: $v_{0},v_{1},\ldots ,v_{n},\ldots$.*
*2*. *Auxiliary symbols: left and right parenthesis, and commas.*
*3*. *Propositional connectives: ¬, ∨, ∧, →,…*
*4*. *Equality symbol: =.*
*5*. *Existential quantifier:* ∃.
*6*. *The symbols of τ.*

The symbols contained in 1 to 4 above are usually referred to as the canonical interpretation and are called logical symbols; the symbols contained in 5 and 6 may have a variable interpretation, and are called non-logical symbols. This is the reason why τ determines the type of language.

Some remarks are in order. In the quantum formalism, a natural choice is to consider the orthogonal projections as individual variables. In classical logic, it is usual to work with only two connectives, as for example, ¬ and ∨, and to define the rest of the connectives as a function of them. This is usual in proof theory to simplify the object language. The possibility of doing this simplification depends on the specific properties of the given language (i.e., connectives are not always inter-definable). In this work, we will restrict the use to ∨, ¬ and ∧. Regarding point 4 above: languages containing this symbol are called languages with an equality. Regarding 5: we will not use quantifiers in this work for the quantum case.

Now we give the standard definitions of terms, expressions and formulas.

**Definition** **7.** *Let τ be a type. An expression of type τ, or a τ-expression, is a finite sequence of symbols of $L_{\tau }$.*

**Definition** **8.** *The set of terms of type τ, or τ-terms, is the least set X of τ-expressions satisfying:*

- *$\{v_{i}\;:\;i\geq 0\}\cup C\subseteq X$, where C⊆τ, is the set of constants of τ.*
- *If $f\in F_{m}\subseteq \tau$, 1≤m and $t_{1},\ldots ,t_{m}\in X$, then $f(t_{1},\ldots ,t_{m})\in X$.*

**Definition** **9.** *A τ-formula is atomic if it is an expression of the form:*

*$\left(t_{1}=t_{2}\right)$ or $P(t_{1},...,t_{n})$, where $t_{1},...,t_{n}$ are τ-terms and $P\in R_{n}\subseteq \tau$.*

**Definition** **10.** *The set of formulas of type τ is the least set X of τ-expressions, such that:*

- *{α:αis an atomicτ-formula}⊆X.*
- *If α,β∈X, then (¬α), (α∨β), (α∧β) and (α→β)∈X.*
- *If α∈X and $v_{i}$ is a variable, then $(\exists v_{i}\alpha )\in X$.*

**Definition** **11.** *A τ-interpretation (or τ-structure) for a language L is a pair U=〈A,I〉, where:*

- *A≠∅.*
- *$I:\tau \to A\cup \left\{f:A^{m}\to A,1\leq m\right\}\cup \left(⋃\left\{P\left(A^{n}\right):1\leq n\right\}\right)$.*

*$P(A^{n})$ denotes the power set of $A^{n}$, and such that for any x∈τ:*

*If $x\in R_{n}$, then $I\left(x\right)=x^{U}\subseteq A^{n}$.*

*If $x\in F_{m}$, then $I\left(x\right)=x^{U}:A^{m}\to A$.*

*If x∈C, then $I\left(x\right)=x^{U}\in A$.*

*I is the interpretation function of U over the universe A.*

Usually, if $\tau =\{x_{1},...,x_{n}\}$, we denote the structure U=〈A,I〉 as follows:

$$U=\langle A,x_{1}^{U},...,x_{n}^{U}\rangle$$

In our case, where the language is formed by projection operators acting on a Hilbert space endowed with their respective connectives, the structure U will have a universe *A* and interpretations for the connectives, which are symbols of functions. In such a structure there will be no interpreted relation symbols. For this reason, the structure associated with our language is an algebra (and not a more general structure), and we can relate it with algebras of the same type as we did in defining valuations and homomorphisms for the language of our type.

**Definition** **12.** *Let U=〈A,I〉 and V=〈B,J〉 be two τ-structures. Then, h is a homomorphism from U to V if, and only if:*

- *h is a function from A to B; h:A→B.*
- *For each relational n-ary symbol $r_{n}$ in τ and each $a_{1},\ldots ,a_{n}\in A$,*
  $$\langle a_{1},\ldots ,a_{n}\rangle \in r_{n}^{U}\;iff\;\langle h\left(a_{1}\right),\ldots ,h\left(a_{n}\right)\rangle \in r_{n}^{V}.$$
- *For each functional n-ary symbol $f_{n}$ in τ and each $a_{1},\ldots ,a_{n}\in A$,*
  $$h\left(f_{n}^{U}\left(a_{1},...,a_{n}\right)\right)=f_{n}^{V}\left(h\left(a_{1}\right),\ldots ,h\left(a_{n}\right)\right).$$
- *For each $c\in \tau ,h\left(c^{U}\right)=c^{V}.$*

It can be seen how this definition for homomorphisms between structures generalizes the one that we gave for a homomorphism between algebras. This is a consequence of the fact that we are dealing with a language of type τ that only has function symbols (which are the connectives of our language).

## 3. Quantum States and the Gleason and Kochen–Specker Theorems

In this section, we review two of the most important results in the foundations of quantum mechanics: Gleason [46,47] and Kochen–Specker [6,8,9] theorems. The fundamental properties underlying these theorems will play a key role in the rest of the paper. We discuss truth functionality in the quantum domain.

### 3.1. Quantum Probabilities and Gleason’S Theorem

An elementary experiment associated with a quantum system is given by a yes-no test, i.e., a test in which we get the answer “yes” or the answer “no” [27]. As is well known, elementary tests associated with quantum systems are represented by orthogonal projections acting on a separable Hilbert space H. An operator P∈B(H) is said to be an orthogonal projection if it satisfies

(1) $$P^{2}=P\;\mathrm{and}\;P=P^{*}.$$

Let P(H) denote the set of all orthogonal projections acting on H. Denote by B(H) the set of bounded operators acting on H. Projectors and closed subspaces can be put in a one to one correspondence, by assigning to each orthogonal projection its image. Thus, they can be considered as interchangeable notions (and we will use them interchangeably in the following). The set P(H) can be endowed with an orthocomplemented lattice structure L(H)=〈P(H),∧,∨,¬,0,1〉, where P∧Q is the orthogonal projection associated to intersection of the ranges of *P* and *Q*, P∨Q is the orthogonal projection associated with the closure of the direct sum of the ranges of *P* and *Q*, 0 is the null operator (the bottom element of the lattice), 1 is the identity operator (the top element), and ¬(P) is the orthogonal projection associated with the orthogonal complement of the range of *P* [31]. It is important to remark that the symbol “L(H)” denotes the collection of orthogonal projections of H and it should not be confused with the set of linear operators acting on it. This lattice (which is equivalent to that of closed subspaces of H) was discovered by Birkhoff and von Neumann, who coined the term Quantum Logic [23]. The main characteristic of this quantum structure is that it is a non-Boolean lattice. It is always modular in the finite-dimensional case and never modular in the infinite one [31].

Any observable quantity can be represented by a self-adjoint operator. For every self-adjoint operator *A*, if the system is prepared in state ρ, its mean value is given by the formula:

(2) 〈A〉=tr(ρA).

Due to the spectral theorem, self-adjoint operators are in one to one correspondence with projective valued measures (PVM) [48]. Let B be the Borel set of the real line. Given a self-adjoint operator *A*, its projection-valued measure $M_{A}$ is a map [31]:

(3) $$M_{A}:B\mapsto P\left(H\right),$$

such that

1. $M_{A}\left(\emptyset \right)=0$
2. $M_{A}\left(R\right)=1$
3. $M_{A}\left(\cup_{j}\left(B_{j}\right)\right)=\sum_{j}M_{A}\left(B_{j}\right)$, for any mutually disjoint family $B_{j}$
4. $M_{A}\left(B^{c}\right)=1-M_{A}\left(B\right)={\left(M_{A}\left(B\right)\right)}^{⟂}$.

In quantum mechanics, states can be considered as functions that assign probabilities to the elements of L(H). A state on a quantum system is represented by a function:

(4) μ:L(H)⟶[0,1]

satisfying:

1. μ(0)=0.
2. $\mu \left(P^{⟂}\right)=1-\mu \left(P\right)$
3. For any pairwise orthogonal and denumerable family ${\left\{P_{j}\right\}}_{j\in N}$, $\mu \left({⋁}_{j}P_{j}\right)=\sum_{j}\mu \left(P_{j}\right)$.

Gleason’s theorem [46,47] is equivalent to the assertion that whenever dim(H)≥3, the set C(L(H)) of all measures of the form (4) can be put in a one to one correspondence with the set S(H) of all positive and trace-class operators of trace one acting in H. The correspondence is such that for every measure μ satisfying the above axioms, there exists a density operator $\rho_{\mu }\in S\left(H\right)$ such that for every orthogonal projector *P* representing an elementary test, we have:

(5) $$\mu \left(P\right)=\mathrm{tr}\left(\rho_{\mu }P\right).$$

It will be important for us to recall how probabilities are defined in a classical setting. Thus, given a set Ω, let us consider a σ-algebra Σ⊆P(Ω) of subsets. Then, a Kolmogorovian probability measure will be given by a function

(6) μ:Σ→[0,1]

satisfying

1. μ(∅)=0
2. $\mu \left(A^{c}\right)=1-\mu \left(A\right),\;\;\mathrm{where}\;{\left(\ldots \right)}^{c}\;\mathrm{denotes}\;\mathrm{set}\;\mathrm{theoretical}\;\mathrm{complement}$
3. for any pairwise disjoint and denumerable family ${\left\{A_{i}\right\}}_{i\in N}$, $\mu \left({⋃}_{i}A_{i}\right)=\sum_{i}\mu \left(A_{i}\right)$.

The above conditions (6) are known as Kolmogorov’s axioms [49] and are very useful for theoretical purposes. The main difference between classical and quantum probabilities—pass their similitude in shape—comes from the fact that the σ-algebra Σ appearing in (6) is Boolean, while L(H) is not. This is the reason why quantum probabilities are also called non-Kolmogorovian (or non-Boolean) probability measures (for more discussion on this subject, see for example [50,51,52,53]; for the connection between quantum probabilities and quantum information theory, see [54,55]).

The lattice L(H) of closed subspaces (or equivalently, orthogonal projections) of a separable Hilbert space and the Boolean algebras (such as those appearing in 6), are all examples of the more general family of orthomodular lattices [56]. A lattice L is said to be orthomodular (or weak modular) if it is orthocomplemented and, whenever x≤y, then $y=x\vee (y\wedge x^{⟂})$. It turns out that the orthogonal projections associated to a von Neumann algebra form an orthomodular lattice [31]. Von Neumann algebras play a key role in the rigorous treatment of quantum systems with infinitely many degrees of freedom [51]. Boolean algebras—such as those appearing in 6—are particular cases of orthomodular lattices. Thus, we can conceive the state of a general physical system described by a measure over an arbitrary complete orthomodular lattice L as follows:

(7) μ:L→[0,1]

which satisfies

1. μ(0)=0
2. $\mu \left(a^{⟂}\right)=1-\mu \left(a\right),\;\;\mathrm{where}\;{\left(\ldots \right)}^{⟂}\;\mathrm{denotes}\;\mathrm{the}\;\mathrm{orthocomplement}$
3. for any pairwise orthogonal and denumerable family of elements ${\left\{a_{i}\right\}}_{i\in I}$, $\mu \left({⋁}_{i}a_{i}\right)=\sum_{i}\mu \left(a_{i}\right)$.

When L is Boolean, we obtain a classical probabilistic model. The lattice L(H) is a particular case among a vast family of alternative models of physical systems.

As a consequence of the orthomodular law, it trivially follows that for every p,q∈L and every state μ, we have p∧q≤p⇒μ(p∧q)≤μ(p) and p≤p∨q⇒μ(p)≤μ(p∨q). These simple inequalities will be used in Section 5 in order to build N-matrices for quantum and generalized probabilistic models.

To finish this section, let us recall an important property of orthomodular lattices that are useful to keep in mind in the following. Any orthomodular lattice—even if it is non-Boolean—possesses Boolean subalgebras [56]. A state defined by Equations (7), when restricted to a maximal Boolean subalgebra, defines a Kolmogorovian probability measure satisfying (6). Moreover, in the quantum formalism, a maximal observable *A* defines a maximal Boolean subalgebra $B_{A}\subseteq L\left(H\right)$, which is given by the range of $M_{A}$ [27].

### 3.2. The Kochen–Specker Theorem and the Failure of Truth Functionality in Quantum Mechanics

The Kochen–Specker theorem is one of the cornerstones in the foundations of quantum mechanics literature [6]. Kochen and Specker were looking for a description of quantum mechanics in terms of hidden variables, taking as a model the relationship between classical statistical mechanics and thermodynamics. It turns out that this hidden variable theory cannot exist for the quantum case. In order to understand the mathematical structures behind the KS theorem, let us define first:

**Definition** **13.** *A classically truth-valued function, is a function v:L(H)⟶{0,1} having the property that $\sum_{i}v\left(P_{i}\right)=1$ for any family ${\left\{P_{i}\right\}}_{i\in N}$ of one dimensional orthogonal elements of L(H) satisfying $\sum_{i}P_{i}=1$.*

The no-go theorem for the hidden variables described in the KS paper is equivalent to the following statement [6].

**Proposition** **1.** *There exists no classically truth-valued function.*

In order to understand what the KS theorem says from a logical point of view, let us see how the notion of truth functionality can be conceived in the quantum domain. Let us identify the quantum language with that of the lattice structure L(H)=〈P(H),∨,∧,¬,0,1〉. It is clear that we can form, recursively, new propositions out of any given set of propositions in the usual way (i.e., consider all possible finite expressions such as (P∨Q)∧R, (¬P∧Q), and so on). If we want to recreate the notion of classical valuation in quantum theory, there should exist a set of functions v:L(H)⟶{0,1} assigning truth values to all possible elementary propositions. In this way, we would have that, given a quantum system prepared in a particular state, all of its properties should satisfy being true or false, and there should be no other possibility. From the physical point of view, there are several extra conditions to be imposed on these classical valuations. For example, if no other restrictions are imposed, one can have the valuation v(P)=0 for all *P*, which is nonsensical because it would imply that all outcomes will have zero probability of occurrence in any experiment. Which restrictions are to be imposed? We need to define a set of conditions in order to discard the non-physical valuations.

According to the probabilistic rules of quantum mechanics (and any physically consistent theory), if proposition *P* is true (i.e., if v(P)=1), then, any other proposition *Q* satisfying $Q\leq P^{⟂}$ should be false (v(Q)=0). This follows directly from the definition of quantum state: if *P* is true, its probability of occurrence is equal to one, and the probability of occurrence of any orthogonal property will be automatically zero (this follows directly from Equation (4); a similar conclusion holds in generalized models by using Equation (7)).

Another condition to be imposed in order to obtain properly classical valuations, should be that of being an algebra homomorphism between L(H) and the two-element Boolean algebra $B_{2}=\left\{0,1\right\}$ (see Definition 3). Thus, for every *P* and every family ${\left\{P_{i}\right\}}_{i=1}^{n}$, in analogy with the principle of truth functionality given by Definition 4, we should have:

(8) $$v\left(\underset{i}{⋁}P_{i}\right)={\tilde{⋁}}_{i}v\left(P_{i}\right)$$

(9) $$v\left(\underset{i}{⋀}P_{i}\right)={\tilde{⋀}}_{i}v\left(P_{i}\right)$$

(10) $$v\left(\neg P\right)=\tilde{\neg }v\left(P\right)=1-v\left(P\right)$$

We call classical admissible valuations (and denote it by CAV) the set of two-valued functions satisfying Equations (8)–(10). Notice that, if the set of admissible valuations is assumed to be CAV, truth-functionality is automatically satisfied.

The condition represented by Equation (10) implies that for a denumerable orthonormal and complete set of projections $P_{i}$ (i.e., $\sum_{i}P_{i}=1$, $P_{i}P_{j}=0$ and $\dim(P_{i})=1$), if $v(P_{i_{0}})=1$ for some $i_{0}$, then, $v(P_{j})=0$, for all $j\neq i_{0}$ (this follows from the fact that for $j\neq i_{0}$, $P_{j}\leq 1-P_{i_{0}}$). Given that $\sum_{i}P_{i}=1$, we must have $v(P_{i_{0}})=1$, for some $i_{0}$ (this follows from v(1)=1 and Equation (8)). This is a very reasonable physical property. It implies that, if an experiment is performed on the system (remember that any denumerable orthonormal and complete set of one-dimensional projections defines an experiment), we obtain that one, and only one projection operator is assigned the value 1, while all other outcomes (which define exclusive events), are assigned the value 0. Notice that this is the condition of KS (i.e., the condition appearing in Definition 13). It is important to remark that an experiment in which all propositions are assigned the value false, or an experiment in which more than one exclusive alternatives are assigned the value true, are physically nonsensical. In the latter case, we would obtain that two mutually exclusive alternatives would occur with complete certainty. In the former, we would reach a situation in which all outcomes of an experiment is false. From the perspective of a classical ontology, these alternatives should be discarded.

We have seen above that any admissible valuation in CAV should satisfy Definition 13. The existence of such functions is strictly forbidden by the KS theorem. It follows that two-valued functions satisfying both, physically reasonable requirements and truth functionality, cannot exist in the quantum domain. This will be naturally true for arbitrary probabilistic models, provided that their propositional structures satisfy the KS theorem (and this is quite true for a huge family of models [7,8,9]). Notice that the KS argument works for Hilbert spaces of dimension greater than or equal to 3. A simple check also shows that no element in CAV exists for a qubit system (i.e., a two-dimensional quantum model). This is so because conditions (8)–(10) are even stronger than those involved in Definition 13.

What about weaker conditions? Perhaps, if we give up some physical considerations and focus only on pure mathematics, we might find a set of admissible functions with regard to which the connectives behave truth functionally. In [57], Friedman, and Glymour study which are the most reasonable conditions to be imposed on the set of admissible valuations. After discarding non-physical possibilities, they end up with the conditions:

v:P(H)⟶{0,1} is an admissible valuation iff

- for every *P*, v(P)=1 iff v(¬P)=0
- for every pair P,Q∈P(H), if v(P)=1 and P≤Q, then v(Q)=1.

Let us call S3 the set of valuations satisfying the above-defined conditions. In [57], S3 is used to denote the list of conditions, but this small difference in notation should not lead to confusion here. Notice first that the conditions that define CAV (i.e., Equations (8)–(10)), imply those that define S3, and thus are stronger. In order to study whether S3 is a non-empty set or not, Friedman and Glymour make further distinctions. First, they consider the normal admissible valuations, which are those satisfying S3, plus the condition that they assign the truth value 1 to at least one one-dimensional subspace. Again, a valuation not satisfying this minimal requirement cannot belong to the realm of a classical ontology. Let us denote by NS3 the set of normal valuations. Clearly, NS3⊆S3. Friedman and Glymour show—by construction—that NS3 is non-empty. Later, they discuss whether it is possible that the valuations in S3 satisfy the minimal physical requirement of realism that every observable have a precise value. For this to happen, it should be the case that for every orthogonal basis, exactly one vector receives the value true and the remainder receive the value false. Let us call RS3 the set of normal admissible functions satisfying this physical requirement. Notice that RS3⊆NS3⊆S3. Again, Friedman and Glymour remark that RS3 is an empty set, due to the KS theorem. By the above discussion, we also have that the conditions defining CAV (Equations (8)–(10)) imply those of RS3, but the converse is not true.

What is left? We are only left then with the functions in NS3, that satisfy the undesirable condition that some observables do not have one (and only one) true projection operator. The whole program of having a reasonable classical valuation satisfying reasonable physical conditions is lost (due to the KS theorem). Even so, if we only restrict to the purely mathematical entities in NS3, G. Hellman showed in [58] that the quantum logical connectives do not behave truth-functionally with regard to those admissible functions. One of the admitted conclusions of Hellman’s work is that his theorem belongs to pure mathematics, with no connection to quantum mechanics.

All roads lead to the same conclusion: it is not possible to define classical truth-functional valuations satisfying reasonable physical requirements (in the sense of CAV and RS3), given that the KS theorem blocks their existence. If one gives up the minimal physical requirements of a classical ontology (using for example, NS3), these functions will not be truth-functional either.

Some important remarks are at stake. The above discussion is related to a well-known fact: it is possible to define local classical valuations for maximal Boolean subalgebras of L(H), but the Kochen-Specker theorem forbids the existence of global ones (see sections II and III of [6]; for more discussion on the subject, see [59,60,61,62,63]). Another related fact is that of the non-existence of states whose range is equal to the set {0,1} (also known as dispersion-free states in the foundations of quantum physics literature). There are several proofs of the non-existence of dispersion free-states, and the KS and Gleason’s theorems can be considered among them. As far as we know, the oldest one is due to J. von Neumann [48], and it is very important to mention here the works of J. Bell on the subject [64,65]. All these works involve different assumptions and mathematical techniques. In this work, we have put the focus on the works of Kochen and Specker, because their approach is the one best fitting to the algebraic structures that we use in order to build the N-matrices for quantum and generalized probabilistic models.

Indeed, if one looks carefully at the KS theorem, it is possible to recognize that a very particular form of the principle of truth functionality fails in the quantum realm. Namely, that there is no classical truth-value assignment *v* satisfying the functionality condition given in Definition 13 (or equivalently, the conditions (8)–(10)). Indeed, in the KS paper [6] (see also [59]), it is proved that this condition underlies the failure of a more general property. In order to illustrate the idea, suppose that the observable represented by the self-adjoint operator *A* has associated the real value of *a*. Then, the observable represented by $A^{2}$, should have assigned the value $a^{2}$. In this sense, observables are not all independent, and neither are the values assigned to them. This gives us a clue for understanding why truth-functionality is not valid in the quantum domain. Following the spirit of the KS paper, let us define:

**Definition** **14.** *Let A(H) be the set of all self-adjoint operators acting on a separable Hilbert space H. A function f:A(H)⟶R satisfies truth functionality if, for any Borel function g:A(H)⟶A(H), if g(A) is the result of applying the function g to a self-adjoint operator A in the usual way, and $f_{X}$ is the result of applying f to an arbitrary self-adjoint operator X, the condition $f_{g(A)}=g\left(f_{A}\right)$ is satisfied.*

A function such as *f* in Definition 14 can be called a prediction function (see section II in [6]) because it assigns a given value to each quantum observable. Condition

(11) $$f_{g(A)}=g\left(f_{A}\right)\;\;,\;\;\mathrm{for}\;\mathrm{every}\;\mathrm{Borel}\;\mathrm{function}\;g,$$

imposes a strong condition on *f*. Let us see how this works (see also sections I and II in [6]). As is well known, two quantum mechanical observables represented by self-adjoint operators *A* and *B*, respectively, are compatible, if and only if, there exist Borel functions $g_{1}$ and $g_{2}$, and a self adjoint operator *C*, such that $A=g_{1}\left(C\right)$ and $B=g_{2}\left(C\right)$. Thus, whenever *A* and *B* are compatible, using the functionality condition (14) we have $f_{AB}=f_{g_{1}\left(C\right)g_{2}\left(C\right)}=f_{\left(g_{1}g_{2}\right)\left(C\right)}=\left(g_{1}g_{2}\right)\left(f_{C}\right)=g_{1}\left(f_{C}\right)g_{2}\left(f_{C}\right)=f_{g_{1}\left(C\right)}f_{g_{2}\left(C\right)}=f_{A}f_{B}$. If α and β are real numbers, we also have, for compatible *A* and *B*, that $f_{\alpha A+\beta B}=f_{\alpha g_{1}\left(C\right)+\beta g_{2}\left(C\right)}=f_{\left(\alpha g_{1}+\beta g_{2}\right)\left(C\right)}=\left(\alpha g_{1}+\beta g_{2}\right)\left(f_{C}\right)=\alpha g_{1}\left(f_{C}\right)+\beta g_{2}\left(f_{C}\right)=\alpha f_{g_{1}\left(C\right)}+\beta f_{g_{2}\left(C\right)}=\alpha f_{A}+\beta f_{B}$. It follows that *f* is a partial Boolean algebra homomorphism. Furthermore, if $P^{2}=P$ and $P^{†}=P$ (i.e., if *P* is an orthogonal projection), we have $f_{P}=f_{P^{2}}=f_{P}f_{P}=f_{P}^{2}$, and then, $f_{P}=0$ or $f_{P}=1$. It should be clear that a function with these properties cannot exist because it goes against Proposition 1 (see also Definition 13). Thus, we see that the failure of the above described sui generis version of truth functionality (the one contained in Definition 14 and Equation (11)), is one of the key features of quantum mechanics. This is true for more general probabilistic models provided that their propositional structures admit no global classical valuations to $B_{2}$ (i.e., provided that they do not admit valuations satisfying Equations (8)–(10)).

In the following sections, we exploit the failure of truth functionality in quantum mechanics and import into physics the solution given by logicians to its equivalent failure in logical systems: we will connect the semantics of N-matrix approach in logic with the formalism of quantum mechanics.

## 4. Non-Deterministic Semantics

Non-deterministic multi-valued matrices (N-matrices) are a fruitful and quickly expanding field of research introduced in [3,4,5] (see also [66,67]). Since then it has been rapidly developing towards a foundational logical theory and has found numerous applications [1]. The novelty of N-matrices is in extending the usual many-valued algebraic semantics of logical systems by importing the idea of non-deterministic computations, and allowing the truth-value of a formula to be chosen non-deterministically out of a given set of options. N-matrices have proved to be a powerful tool, the use of which preserves all the advantages of ordinary many-valued matrices, but is applicable to a much wider range of logic. Indeed, there are many useful (propositional) non-classical logics, which have no finite multi-valued characteristic matrices, but do have finite N-matrices, and thus are decidable.

### 4.1. Deterministic Matrices

Here we follow the presentation of the subject given in [1]. In what follows, *L* is a propositional language and $Frm_{L}$ is its set of well formed formulas. The metavariables φ, ψ, …, range over *L*-formulas, and Γ, Δ, …, over sets of *L*-formulas. The standard general method for defining propositional logics is by using (possibly many-valued) deterministic matrices:

**Definition** **15.** *A matrix for L is a tuple*

P=〈V;D;O〉,

*where*

- *V is a non-empty set of truth-values.*
- *D (designated truth-values) is a non-empty proper subset of V.*
- *For every n-ary connective ⟡ of L, O includes a corresponding function $\tilde{⟡}:V^{n}\to V$*

*A partial valuation in P is a function v to V from some subset $W\subseteq Frm_{L}$ which is closed under subformulas, such that for each n-ary connective ⟡ of L, the following holds for all $\psi_{1},...,\psi_{n}\in W:$*

(12) $$v\left(⟡\left(\psi_{1},...,\psi_{n}\right)\right)=\tilde{⟡}\left(v\left(\psi_{1}\right),...,v\left(\psi_{n}\right)\right)$$

### 4.2. Non-Deterministic Matrices (N-Matrices)

Now we turn into the non-deterministic case. The main difference is that, alike deterministic matrices, the non-deterministic ones, given the inputs in the truth table, assign a set of possible values instead of a single one.

**Definition** **16.** *A non-deterministic matrix (N-matrix) for L is a tuple M=〈V,D,O〉, where:*

- *V is a non-empty set of truth-values.*
- *D∈P(V) (designated truth-values) is a non-empty proper subset of V.*
- *For every n-ary connective ⟡ of L, O includes a corresponding function*
  $$\tilde{⟡}:V^{n}\to P\left(V\right)\setminus \left\{\emptyset \right\}$$

**Definition** **17.**

*1*. *A partial dynamic valuation in M (or an M-legal partial dynamic valuation), is a function v from some closed under subformulas subset $W\subseteq Frm_{L}$ to V, such that for each n-ary connective ⟡ of L, the following holds for all $\psi_{1},\ldots ,\psi_{n}\in W$:*
  $$v\left(⟡\left(\psi_{1},\ldots ,\psi_{n}\right)\right)\in \tilde{⟡}\left(v\left(\psi_{1}\right),\ldots ,v\left(\psi_{n}\right)\right).$$
*A partial valuation in M is called a valuation if its domain is $Frm_{L}$.*
*2*. *A (partial) static valuation in M (or an M-legal (partial) static valuation), is a (partial) dynamic valuation (defined in some $W\subseteq Frm_{L}$) which satisfies also the following composability (or functionality) principle: for each n-ary connective ⟡ of L and for every $\psi_{1},\ldots ,\psi_{n},\varphi_{1},\ldots ,\varphi_{n}\in W$, if $v\left(\psi_{i}\right)=v\left(\varphi_{i}\right)\;\;\left(i=1,\ldots ,n\right)$, then*
  $$v\left(⟡\left(\psi_{1},\ldots ,\psi_{n}\right)\right)=v\left(⟡\left(\varphi_{1},\ldots ,\varphi_{n}\right)\right).$$

It is important to remark that ordinary (deterministic) matrices correspond to the case when each $\tilde{⟡}:V^{n}\to V$ is a function taking singleton values only. In this case there is no difference between static and dynamic valuations, and we have full determinism.

To understand the difference between ordinary matrices and N-matrices, recall that in the deterministic case, the truth-value assigned by a valuation *v* to a complex formula is defined as follows: $v\left(⟡\left(\psi_{1},...,\psi_{n}\right)\right)=\tilde{⟡}\left(v\left(\psi_{1}\right),...,v\left(\psi_{n}\right)\right)$. Thus the truth-value assigned to $⟡(\psi_{1},...,\psi_{n})$ is uniquely determined by the truth-values of its subformulas: $v\left(\psi_{1}\right),...,v\left(\psi_{n}\right)$. This, however, is not the case for N-matrices: in general, the truth-values of $\psi_{1},...,\psi_{n}$, do not uniquely determine the truth-value assigned to $⟡(\psi_{1},...,\psi_{n})$ because different valuations having the same truth-values for $\psi_{1},...,\psi_{n}$ can assign different elements of the set of options $\tilde{⟡}\left(v\left(\psi_{1}\right),...,v\left(\psi_{n}\right)\right)$ to $⟡(\psi_{1},...,\psi_{n})$. Therefore the non-deterministic semantics is non-truth-functional, as opposed to the deterministic one. Notice also that the indeterminism that appears in the context of N-matrices is defined in terms of the behavior of valuations, and is related to the failure of truth-functionality. This notion should not be confused with the non-deterministic character of quantum phenomena. Whether these notions can be connected or not, will be the subject of future work. In Table 1, we sketch the differences between deterministic and non-deterministic matrices. Now, we review the standard definitions of logical consequence [1].

**Definition** **18.**

*1*. *A (partial) valuation v in M satisfies a formula ψ (v⊧ψ) if (v(ψ) is defined and) v(ψ)∈D. It is a model of Γ (v⊧Γ) if it satisfies every formula in* Γ.
*2*. *We say that ψ is dynamically (statically) valid in M, in symbols ${⊧}_{M}^{d}\psi$ (${⊧}_{M}^{s}\psi$), if v⊧ψ for each dynamic (static) valuation v in M.*
*3*. *the dynamic (static) consequence relation induced by M is defined as follows: $\Gamma {⊢}_{M}^{d}\Delta$ ($\Gamma {⊢}_{M}^{s}\Delta$) if every dynamic (static) model v in M of *Γ* satisfies some ψ∈Δ.*

Obviously, the static consequence relation includes the dynamic one, i.e., ${⊢}_{M}^{d}\subseteq {⊢}_{M}^{s}$. For ordinary matrices, we have ${⊢}_{M}^{s}={⊢}_{M}^{d}$.

**Theorem** **1.** *Let M be a two-valued N-matrix which has at least one proper non-deterministic operation. Then there is no finite family of finite ordinary matrices F such that ${⊢}_{M}^{d}\psi \;\mathrm{iff}\;{⊢}_{F}\psi$.*

**Theorem** **2.** *For every (finite) N-matrix M, there is a (finite) family of ordinary matrices F such that ${⊢}_{M}^{s}={⊢}_{F}$.*

Thus, only the expressive power of the dynamic semantics based on N-matrices is stronger than that of ordinary matrices.

## 5. N-Matrices for Probabilistic Theories

As we have seen in Section 3, the Kochen-Specker theorem forbids the existence of a homomorphism (i.e., valuations satisfying Equations (8)–(10)) from the lattice of quantum propositions to the two-valued algebra $B_{2}$. There, we showed how one of the most important presuppositions of the Kochen-Specker contradiction can be related to the logical notion of truth functionality. Weaker versions of admissible valuations also fail to give a truth-functional system. This is the case, for example, of the valuations contained in the set NS3. Given that valuations in a semantics-based in non-deterministic matrices are not, in general, truth-functional, they could provide an interesting way of describing those formal aspects of quantum theory related to Kochen–Specker-like contextuality. Thus, given that quantum states cannot be interpreted in terms of classical (deterministic) valuations (i.e., valuations in CAV), in this section we aim to describe them as valuations of a non-deterministic semantics. It turns out that there are several ways to associate non-deterministic matrices to the quantum formalism. Furthermore, we show that quantum states can be described as valuations associated with a very particular form of non-deterministic truth tables.

### 5.1. Construction of the N-Matrices for the Quantum Formalism

In this section, we build the canonical N-matrices of the quantum formalism. We use the lattice of propositions L(H) appearing in (4) and the physical constraints imposed by the properties of quantum states, in such a way that the valuations defined by our matrices are exactly those given by quantum states. Let V=[0,1] and D={1}. A proposition will be true if and only if its valuation yields the value 1, and it is false for any other value (this is connected to the standard quantum logical notion of truth; see discussion in [27]). In order to build the matrices, we take into account first that for every state μ, whenever P⟂Q, we have μ(P∨Q)=μ(P)+μ(Q) (this follows from (4)). Let us now study the disjunction function $\tilde{\vee }:V\times V\to P\left(V\right)\setminus \left\{\emptyset \right\}$. From the Equation (4), it is easy to check that max(μ(P),μ(Q))≤μ(P∨Q)≤1. In terms of valuations (denoted generically by *v*), this can be written as

max(v(P),v(Q))≤v(P∨Q)≤1.

In this way, the natural candidate for the disjunction matrix is given by

(13) PQ∨˜if P⊥Qab{a+b}if P⊥Qab[max(a,b);1]

Let us now turn to the functions associated to the conjunction. They have to be of the form $\tilde{\wedge }:V\times V\to P\left(V\right)\setminus \left\{\emptyset \right\}$. Proceeding as before, we obtain that valuations should satisfy

v(P∧Q)≤min(v(P),v(Q)).

Thus, whenever a valuation v(⋯) assigns v(P)=a and v(Q)=b (a,b∈V), it is reasonable to define the following N-matrix:

(14) PQ∧˜if P⊥Qab[0,min(a,b)]if P⊥Qab{0}.

Notice that in the tables (13) and (14) the connectives “∨” and “∧” are defined by parts, in the sense that we distinguish between their restrictions to the case of orthogonal vs. non-orthogonal input propositions. Formally speaking, this means that each Table describes the functions associated with two partially defined connectives (each line of each Table defines a function associated with a partially defined connective).

It remains to give the table for the negation $\tilde{\neg }:V\to P\left(V\right)\setminus \left\{\emptyset \right\}$. The most natural candidate compatible with the properties of quantum states (4) is given by $\tilde{\neg }\left(a\right)=\left\{1-a\right\}$, and then

(15) $$\begin{array}{cc} P & \tilde{\neg }\\ a & \left\{1-a\right\} \end{array}$$

This is a deterministic negation, in the sense that its interpretation set is a singleton, which is a function of *a*.

The above three tables (considered together) impose the restrictions for all possible valuations. A closer look at them reveals that for finite-dimensional models they contain the very conditions of Gleason’s theorem. Thus, the only valuations that satisfy them (for finite-dimensional models) are those defined by quantum states. Let us see how this is so. First, notice that any quantum state will satisfy the three tables. Thus, the set of quantum states is contained in the set of valuations defined by the above tables. Conversely, suppose that a given valuation *v* satisfies the three tables. As a valuation, it will take values in the interval [0,1]. In order to see that condition 1 of (4) is valid, let us first take two arbitrary orthogonal one-dimensional projections *P* and *Q*. Since they are orthogonal, using the truth-table of the conjunction, we have v(P∧Q)=0. On the other hand, given that P∧Q=0 (since they are orthogonal), we conclude that v(0)=v(P∧Q)=0, which is condition 1 of (4). It is also important to remark that if P≤Q, then P∨Q=Q and, using 13 for the case P⊥Q (when P≠0), it follows that v(Q)=v(P∨Q)≥max(v(P),v(Q))≥v(P). Thus, it easily follows that any valuation compatible with the above tables will be *order preserving*. When P⊥Q, we have $P\leq Q^{⊥}$, and then, $v\left(P\right)\leq v\left(Q^{⊥}\right)$. Using 15, this can be rewritten as v(P)≤1-v(Q). Thus, it follows that for P⊥Q, v(P)+v(Q)≤1 (notice that, in the first line of 13, we could have set {min(a+b,1)} instead of the simpler expression {a+b}; but since v(P)+v(Q)≤1 for all *v*, this is not necessary). Whenever two orthogonal projections are given, due to the truth-table of the disjunction, we have v(P∨Q)=v(P)+v(Q). For finite-dimensional models, this is equivalent to condition 3 in (4). Condition 2 of (4) is automatically satisfied due to the truth table of the negation. Thus, we have proved that any valuation satisfying the three tables also satisfies the axioms of quantum states (for finite-dimensional models), and then, it has to be a quantum state. In other words, we have that, for finite-dimensional models, the only valuations that satisfy the above non-deterministic truth-tables are the quantum states (i.e., those states defined by density matrices).

For infinite-dimensional models of quantum systems, one could still try to impose a σ-additivity condition (such as the one appearing in (4)), and apply again Gleason’s theorem to obtain quantum states. This involves the use of a condition on a denumerable set of propositions. We will study this possibility in a future work. It is also important to remark that there are generalized versions of Gleason’s theorem [68] that can be used to study additive (and not necessarily σ-additive) measures, and could be an interesting subject of study in future works.

One last thing remains. According to the above-defined tables, we may ask: are the above tables truth-functional with regard to the quantum logical connectives? To begin with, notice that the negation table is classical. More concretely: is it true that for every two propositions *P* and *Q*, and every quantum logical connective ⋄, if the probabilities assigned to *P* and are *a* and *b*, respectively, the value of the probability of P⋄Q is determined by *a* and *b*? This question is tricky, because, even if the valuations are valued into sets with more than one element, Gleason’s theorem could impose, in principle, restrictions in such a way that all states that assign probabilities *a* and *b* to *P* and *Q*, respectively, assign the same value for the composed proposition P⋄Q. This is explicitly the case when P⊥Q: for all ρ∈S(H), if tr(ρP)=a and tr(ρP)=b, we have that tr(ρ(P∨Q))=a+b and tr(ρ(P∧Q))=0. It turns out that this is not the case when P⊥Q, as the following examples show.

Consider first a four dimensional quantum model and the basis {|a〉,|b〉,|c〉,|d〉}. Consider the propositions defined by the projection operators P=|a〉〈a|+|b〉〈b| and Q=|b〉〈b|+|c〉〈c|. Clearly, R:=P∧Q=|b〉〈b| and P⊥Q. We chose for simplicity α,β,γ,δ∈R and consider the state $|\psi \rangle =\sqrt{\alpha }|a\rangle +\sqrt{\beta }|b\rangle +\sqrt{\gamma }|c\rangle +\sqrt{\delta }|d\rangle$. Thus, the probabilities of the elements of the basis are given by α, β, δ and γ, respectively, and the normalization condition reads α+β+γ+δ=1. A simple calculation yields that the probability of *P* is $p_{\psi }\left(P\right)=\mathrm{tr}(|\psi \rangle \langle \psi |P)=\alpha +\beta$, the probability of *Q* is $p_{\psi }\left(Q\right)=\mathrm{tr}(|\psi \rangle \langle \psi |Q)=\beta +\gamma$ and the probability of *R* is $p_{\psi }\left(P\wedge Q\right)=\mathrm{tr}(|\psi \rangle \langle \psi |\left(P\wedge Q\right))=\beta$. Now, chose 0<ε<α,β,γ,δ, and consider a new quantum state defined by $|\psi_{\varepsilon }\rangle =(\sqrt{\alpha +\varepsilon }|a\rangle +\sqrt{\beta -\varepsilon }|b\rangle +\sqrt{\gamma +\varepsilon }|c\rangle +\sqrt{\delta -\varepsilon }|d\rangle$ (the reader can easily check that the normalization is correct). Now, we have that the probability of *P* is $p_{\psi_{\varepsilon }}\left(P\right)=\mathrm{tr}(|\psi_{\varepsilon }\rangle \langle \psi_{\varepsilon }|P)=\alpha +\varepsilon +\beta -\varepsilon =\alpha +\beta$, the probability of *Q* is $p_{\psi_{\varepsilon }}\left(Q\right)=\mathrm{tr}(|\psi_{\varepsilon }\rangle \langle \psi_{\varepsilon }|Q)=\beta -\varepsilon +\gamma +\varepsilon =\beta +\gamma$ and the probability of *R* is $p_{\psi_{\varepsilon }}\left(P\wedge Q\right))=\mathrm{tr}(|\psi_{\varepsilon }\rangle \langle \psi_{\varepsilon }|\left(P\wedge Q\right)=\beta -\varepsilon \neq \beta$. Thus, we have two different states that assign the same probabilities to *P* and *Q*, but different values to P∧Q.

With the same notation as in the previous example, we have that S:=P∨Q=|a〉〈a|+|b〉〈b|+|c〉〈c|. Again, we have P⊥Q, $p_{\psi }\left(P\right)=\alpha +\beta$ and $p_{\psi }\left(Q\right)=\beta +\gamma$. For the disjunction, we now have $p_{\psi }\left(P\vee Q\right)=\mathrm{tr}(|\psi \rangle \langle \psi |\left(P\vee Q\right))=\alpha +\beta +\gamma$. Computing the probabilities for state $|\psi_{\varepsilon }\rangle$, we obtain again $p_{\psi_{\varepsilon }}\left(P\right)=\alpha +\beta$ and $p_{\psi_{\varepsilon }}\left(Q\right)=\beta +\gamma$. The probability of the disjunction is given by $p_{\psi_{\varepsilon }}\left(P\vee Q\right)=\mathrm{tr}(|\psi_{\varepsilon }\rangle \langle \psi_{\varepsilon }|\left(P\vee Q\right)=\alpha +\varepsilon +\beta -\varepsilon +\gamma +\varepsilon =\alpha +\beta +\gamma +\varepsilon \neq \alpha +\beta +\gamma$. Thus, we have two different states that assign the same probabilities to *P* and *Q*, but different values to P∨Q.

These examples show that the truth tables defined above define a strictly non-deterministic semantics: the valuations which are compatible with those tables are dynamic. Are they static? The following example shows that this is not the case.

Consider a three dimensional Hilbert space with a basis {|a〉,|b〉,|c〉}. Define P=|a〉〈a| and Q=|φ〉〈φ| (where $|\varphi \rangle =\frac{1}{\sqrt{2}}(|a\rangle +\left|b\rangle \right)$)). The conjunction is given by P∨Q=|a〉〈a|+|b〉〈b| (due to the fact that they are two linearly independent vectors, they define a closed subspace of dimension 2). Consider the state |ϕ〉=|b〉. Thus, we have $p_{\phi }\left(P\right)=\mathrm{tr}(|b\rangle \langle b|a\rangle \langle a|)=0$, $p_{\phi }\left(Q\right)=\frac{1}{2}$ and $p_{\phi }\left(P\vee Q\right)=\mathrm{tr}(|b\rangle \langle b|\left(P\vee Q\right))=\mathrm{tr}(|b\rangle \langle b|(|a\rangle \langle a|+\left|b\rangle \langle b|)\right)=1$. Consider now $P^{\prime }=\left|c\rangle \langle c\right|$ and $Q^{\prime }=Q$. We again have $p_{\phi }\left(P^{\prime }\right)=\mathrm{tr}(|b\rangle \langle b|c\rangle \langle c|)=0$ and $p_{\phi }\left(Q^{\prime }\right)=\frac{1}{2}$. The disjunction is given by $P^{\prime }\vee Q^{\prime }=\left|\varphi \rangle \langle \varphi \right|+\left|c\rangle \langle c\right|$ (we are using that |c〉 and |φ〉 are orthogonal). Now, $p_{\phi }\left(P^{\prime }\vee Q^{\prime }\right)=\mathrm{tr}(|b\rangle \langle b|(|\varphi \rangle \langle \varphi |+|c\rangle \langle c|))=\mathrm{tr}(|b\rangle \langle b|\varphi \rangle \langle \varphi |)+\mathrm{tr}(|b\rangle \langle b|c\rangle \langle c|=\frac{1}{2}+0=\frac{1}{2}\neq 1$. Thus, we have obtained that, given two pairs of propositions, *P* and *Q*, and $P^{\prime }$ and $Q^{\prime }$, there is a valuation $v_{\phi }\left(...\right)=\mathrm{tr}(|\phi \rangle \langle \phi |\left(...\right))$ (induced by the quantum state |ϕ〉), that satisfies $v_{\phi }\left(P\right)=v_{\phi }\left(P^{\prime }\right)$, $v_{\phi }\left(Q\right)=v_{\phi }\left(Q^{\prime }\right)$, but $v_{\phi }\left(P\vee Q\right)\neq v_{\phi }\left(P^{\prime }\vee Q^{\prime }\right)$. Thus, in general, the valuations will not be static. Thus, the valuations defined by quantum states will not be static in general.

In Table 2, we summarize the differences between the classical and quantum cases.

### 5.2. The General Case

In this section, we turn into the non-deterministic matrices for generalized probabilistic models, whose propositional structures are defined by arbitrary complete orthomodular lattices and states are defined by Equation (7). We proceed in a similar way to that of the quantum case, but it is important to take into account that now (a) Gleason’s theorem will no longer be available in many models, and (b) the difference between additivity and σ-additivity imposes a great restriction if one wants to link valuations with states (see [68] for generalizations of Gleason’s theorem and the discussion about the difference between additivity and σ-additivity). In the general case, it will be possible to affirm that every state defines a valuation, but there will exist valuations which are no states. Furthermore, if the lattice admits no states [69], then the matrices that we define will admit no valuations at all.

It is also very important to remark that the N-matrices introduced below, work well when the lattices are Boolean algebras. This means that classical (Kolmogorovian) probabilistic models (defined by Equation (6)) also fall into our scheme. Indeed, following a similar procedure to the one of the preceding section, it is possible to check that the N-matrices associated with a classical probabilistic model are strictly non-deterministic. A similar conclusion can be reached from the point of view of multi-valued logics [70]. Alike the quantum case, classical probabilistic models always admit global classical valuations whose range is equal to the set {0,1} (i.e., they admit valuations obeying Equations (8)–(10)). If the requirements of physics are satisfied, due to the KS and Gleason’s theorems, the range of global valuations associated with the N-matrices associated with quantum systems cannot be equal to {0,1}. A similar observation holds for more general probabilistic models, provided that they are contextual enough and they admit states.

Let us first build the interpretation set for the conjunction function $\tilde{\wedge }:V\times V\to P\left(V\right)\setminus \left\{\emptyset \right\}$. Using Equation (7), it easily follows that μ(p∧q)≤min(μ(p),μ(q)). Remember also that, in the general setting, p⊥q iff $p\leq q^{⊥}$ (or equivalently, $q\leq p^{⊥}$), where ${\left(...\right)}^{⊥}$ is the orthocomplementation in L (see [56], Chapter 1). Thus, whenever a valuation v(⋯) assigns v(p)=a and v(q)=b (a,b∈V), the natural truth table for the conjunction can be given by:

(16) pq∧˜if p⊥qab[0,min(a,b)]ifp⊥qab{0}

Let us now study the disjunction function $\tilde{\vee }:V\times V\to P\left(V\right)\setminus \left\{\emptyset \right\}$. Using (7), we obtain max(μ(p),μ(q))≤μ(p∨q)≤1 and max(v(p),v(q))≤v(p∨q)≤1. Thus, the truth-table is given by:

(17) pq∨˜If p⟂qab{a+b}if p⊥qab[max(a,b);1]

In the general setting, the negation of a proposition *a* is represented by its orthogonal complement “$a^{⟂}$” in the lattice. In order to impose restrictions on valuations, we use (7) (in the following section, we will consider more general possibilities). Thus, we define $\tilde{\neg }\left(a\right)=\left\{1-a\right\}$ and obtain:

(18) $$\begin{array}{cc} p & \tilde{\neg }\\ a & \left\{1-a\right\} \end{array}$$

## 6. Other Logical Aspects of Our Construction

In this section, we turn into other logical aspects of the quantum N-matrices. We start by discussing the notion of adequacy and introduce variants of the deterministic negation defined by (15). Next, we discuss how they behave under the application of double negation. Finally, we discuss the notion of logical consequence defined by the quantum N-matrices. As is well known, the inclusion relationship between projection operators is not—from a logical point of view—a true implication. By constructing a suitable N-matrix semantics for the quantum formalism, we would obtain the benefits of the notion of logical consequence developed by Avron and Zamansky [1]. One of the most important advantages of the N-matrix system is that, given that it is usually represented by finite matrices, it is possible to prove its decidability. In our approach—in order to obtain a closer connection with the quantum formalism—we assumed that *V* is non-denumerable. We show below that it is always possible to reduce the cardinality of *V* to that of a denumerable set, without affecting the set of theorems. This is directly related to the Definitions of F-expansion and refinement (20).

### 6.1. Quantum N-Matrices and Adequacy

Let us now turn to the notion of adequacy of N-matrices:

**Definition** **19.** *Let M=〈V,D,O〉 be an N-matrix for a language which includes the positive fragment of the classical logic ($LK^{+}$). We say that M is adequate for this language, in case that the following conditions are satisfied:*

*1*. *$\tilde{\wedge }$:*
  $$If\;\;\;a\in D\;\;and\;\;b\in D,\;\;then\;\;a\tilde{\wedge }b\subseteq D$$
  $$\mathrm{If}\;\;\;a\notin D,\;\;\mathrm{then}\;\;a\tilde{\wedge }b\subseteq V\setminus D$$
  $$\mathrm{If}\;\;\;b\notin D,\;\;\mathrm{then}\;\;a\tilde{\wedge }b\subseteq V\setminus D$$
*2*. *$\tilde{\vee }$:*
  $$\mathrm{If}\;\;\;a\in D,\;\;\mathrm{then}\;\;a\tilde{\vee }b\subseteq D$$
  $$\mathrm{If}\;\;\;b\in D,\;\;\mathrm{then}\;\;a\tilde{\vee }b\subseteq D$$
  $$\mathrm{If}\;\;\;a\notin D\;\;\mathrm{and}\;\;b\notin D,\;\;\mathrm{then}\;\;a\tilde{\vee }b\subseteq V\setminus D$$
*3*. *$\tilde{⊃}$:*
  $$\mathrm{If}\;\;\;a\notin D,\;\;\mathrm{then}\;\;a\tilde{⊃}b\subseteq D$$
  $$\mathrm{If}\;\;\;b\in D,\;\;\mathrm{then}\;\;a\tilde{⊃}b\subseteq D$$
  $$\mathrm{If}\;\;\;a\in D\;\;\mathrm{and}\;\;b\notin D,\;\;\mathrm{then}\;\;a\tilde{⊃}b\subseteq V\setminus D$$

In this section we briefly discuss Definition 19 in the context of quantum generalized N-matrices with finite precision measurements. By this, we mean a situation in which it is not possible to determine if a proposition is true, but it is possible to assure that it is included within a certain interval around 1. In order to do this, we can now take the same tables defined by (13)–(15) (or equivalently, (17), (16) and (18) for the general case), and change the designated values set to D=[α,1], with α∈(0,1]. By setting α=1, we obtain the matrices of the previous sections.

It is important to remark that with an interpretation set such as D=[α,1] and tables defined by (13)–(15) (or (17), (16) and (18)), it could be the case that a valuation selects a non-designated value, even when both input elements are designated (notice that this is also true for the case α=1). Let us illustrate this with an example. Suppose that α=1 and we prepare a quantum system in a state $|\psi \rangle =\frac{1}{\sqrt{2}}(|\psi_{1}\rangle +|\psi_{2}\rangle )$ with $\langle \psi_{1}|\psi_{2}\rangle =0$. Thus, the valuation associated to the state $\rho_{\psi }=\left|\psi \rangle \langle \psi \right|$, assigns non-designated values to the propositions $|\psi_{1}\rangle \langle \psi_{1}|$ and $|\psi_{2}\rangle \langle \psi_{2}|$, while it assigns a designated value to the proposition $|\psi_{1}\rangle \langle \psi_{1}\left|+\right|\psi_{2}\rangle \langle \psi_{2}|$ (associated to the disjunction $|\psi_{1}\rangle \langle \psi_{1}|$ and $|\psi_{2}\rangle \langle \psi_{2}|$). This makes our matrix non-adequate because it violates condition 2 of Definition 19. Thus, in general, quantum states will not satisfy adequacy. Adequacy is not a requirement for physics, but it could be of interest to logicians. For example, it is possible to use the criterion of Definition 19 to give unicity proofs for matrices given a certain language and conditions. Thus, let us see what can we do in order to obtain an adequate matrix. We must first restrict the interpretation set of the conjunction. Notice that, by doing this, we depart from physics, given that the new valuations may no longer be quantum states.

Let us start by defining the interpretation set for the conjunction $\tilde{\wedge }:V\times V\to P\left(V\right)\setminus \left\{\emptyset \right\}$. As before, *a* and *b* represent the respective values for the valuations of *p* and *q*: v(p)=a and v(q)=b. Let us discuss case by case.

- If a,b∈D:

Given that 0≤v(p∧q)≤min(a,b), this suggests us to take the interpretation set for the conjunction for the case where both values are designated as:

(19) $$a\tilde{\wedge }b\subseteq \left[0,min\left(a,b\right)\right]\cap D$$

- If a∈D, and b∉D

Proceeding similarly as before, we obtain v(p∧q)≤min(a,b), but now we know which is the smallest between *a* and *b*, given that *b* is not designated. Thus,

0≤v(p∧q)≤b

This suggests the following interpretation:

(20) $$a\tilde{\wedge }b\subseteq \left[0,b\right]\subseteq V\setminus D.$$

It is easy to check that this case satisfies the adequacy criterion without the necessity of restricting the set.

The case a∉D and b∈D is totally analogous.

- If a,b∉D:

Given that

0≤v(p∧q)≤min(a,b),thus

(21) $$a\tilde{\wedge }b\subseteq \left[0,\min\left(a,b\right)\right]\subseteq V\setminus D.$$

Proceeding in an analogous way, we now obtain the interpretation set of the disjunction:

$$\tilde{\vee }:V^{2}\to 2^{V}\setminus \left\{\emptyset \right\}$$

- If a,b∈D

Given that p≤p∨q and q≤p∨q, then μ(p)≤μ(p∨q) and μ(q)≤μ(p∨q).

a≤v(p∨q)andb≤v(p∨q),thus

max(a,b)≤v(p∨q)≤1

Thus,

(22) $$a\tilde{\vee }b\subseteq \left[\max\left(a,b\right),1\right]\subseteq D$$

- If a∈D,b∉Dora∉D,b∈D

(23) $$a\tilde{\vee }b\subseteq \left[\max\left(a,b\right),1\right]\subseteq D$$

- If a,b∉D

If none of the two terms has a designated value, one possibility is to proceed as in the first case with the conjunction (by restricting our set in such a way that it satisfies the adequacy criterium).

$$a\tilde{\vee }b\subseteq \left[\max\left(a,b\right),1\right]$$

A valuation for two projections with non-designated values could give us a designated value. If we want to avoid this, and using Avron’s criterion, the interpretation set should be:

(24) $$a\tilde{\vee }b\subseteq \left[\max\left(a,b\right),1\right]\cap \left(V\setminus D\right).$$

We will not follow this choice, given that Gleason’s theorem imposes stronger restrictions on valuations.

Let us turn now to the negation. We consider different choices, alternative to (18). Given that we are working with orthomodular lattices, we have that any state satisfies $\mu \left(p^{⊥}\right)=1-\mu \left(p\right)$. In terms of valuations, this condition reads: $v\left(p^{⊥}\right)=1-v\left(p\right)$. In order to obtain a non-deterministic negation, our first choice is:

Case 1:

(25) $$\begin{array}{cc} p & {\tilde{\neg }}_{1}\\ a\in D & \left[0,1-a\right]\\ a\notin D & \left[1-a,1\right] \end{array}$$

This case generalizes the standard quantum one (given by (15)), leaving 1-a (deterministic negation) as a respective bound.

Case 2: We introduce parameters now. Let us assume that $\alpha \in (\frac{1}{2},1)$ and define:

(26) $$\begin{array}{cc} p & {\tilde{\neg }}_{2}\\ a\in D & \left[\alpha -\frac{a}{2}\left(\frac{1-\alpha }{\alpha }\right),\alpha \right)\\ a\notin D & \left[\alpha ,\alpha +\frac{a}{2}\left(\frac{1-\alpha }{\alpha }\right)\right] \end{array}$$

Both sets depend now on the value of α.

Finally, by appealing to (15) and (18), we can always define deterministic negations. This is perhaps the more natural choice for the standard quantum case and more general probabilistic models. In this case, independently of whether the value of *a* is designated or not, the negation yields 1-a. If we take D={1} as in the standard quantum logical case, then the negation of a given designed value would give a non-designed result. The converse is not necessarily true.

### 6.2. Double Negation

Now we turn to some problems that could emerge with the behavior of the double negation. A more detailed study of the negation and double negation is left for future work. We consider both, logical and physical motivations in order to proceed.

Given its relation to the orthogonal complement in Hilbert spaces, it is desirable that the negation satisfies the principle of double negation. It is obvious that the negations defined in (15) and (18) satisfy this principle. We now show that, despite of not respecting this principle strictly, the negation ${\tilde{\neg }}_{1}$ (see (25)) has a very particular behavior that could be related to the classical limit between logics. Although the negations defined by (25) and (26) do not behave properly in this sense, their existence is interesting on its own, given that, in a different domain, we may need different types of connectives associated with the negation. As an example, in quantum circuits, it is possible to define an operation which is the square root of the negation. It is important to remark that the failure of double negation is also found in other algebraic structures associated with the quantum formalism (see for example [71]).

Let us now analyze the double negation for ${\tilde{\neg }}_{1}$. Applying it twice, we obtain:

$$v(\left(\neg_{1}\left(\neg_{1}\left(p\right)\right)\right)\in {\tilde{\neg }}_{1}\left(v\left(\neg_{1}p\right)\right)$$

$$v\left(\neg_{1}p\right)\in {\tilde{\neg }}_{1}\left(v\left(p\right)\right)$$

Let us analyze the different cases in relation to the set of designated values. We assume that 0.5<α.

- If v(p)=a∈D=[α,1]:

$${\tilde{\neg }}_{1}\left(a\right)=\left[0,1-a\right]\;\mathrm{and}\;v\left(\neg_{1}p\right)\in \left[0,1-a\right]$$

Let $b=v\left(\neg_{1}p\right)\in \left[0,1-a\right]\subseteq \left(V\setminus D\right)$. Then:

$$v\left(\neg_{1}\left(\neg_{1}p\right)\right)\in {\tilde{\neg }}_{1}\left(b\right)=\left[1-b,1\right],$$

given that *b* is not a designated value. Thus, the double negation maps designated values to designated values. If we order all possible values, then:

0≤b≤1-a≤α≤a≤1-b≤1

This proves that, after taking two times the negation of a designated value, the value for the double negation must be chosen out of a set which is included in the set from which the original designated value was taken. This means that, when taking consecutively the double negation for the designated case, the lower bound of the final interpretation set for the connective is closer to {1}. Now we show that this will not happen if we start from a non-designated value.

- If $v_{\left(p\right)}=a\notin D$

$${\tilde{\neg }}_{1}\left(a\right)=\left[1-a,1\right]$$

Let *b* be the value that the valuation takes inside this set:

$$b=v_{\left(\neg_{1}p\right)}\in \left[1-a,1\right]$$

Then, we obtain

$$v\left(\neg_{1}\left(\neg_{1}p\right)\right)\in {\tilde{\neg }}_{1}\left(b\right)$$

The difference with regard to the previous case is that now *b* could be a designated value or not, given that inside the interval [1-a,1], in principle, there could be values of both types. As an example, if α=0.9 and a=0.8, the involved interval would be [0.2,1], which contains both designed and non-designed values. Thus, for the double negation, in this case, we have to separate the interpretation set depending on the type of value taken by *b*.

This behavior was already included in the orthodox quantum logical treatment, where the negation of a true proposition was false, but the negation of something false is not necessarily true. Thus, we obtain:

$$v\left(\neg_{1}\left(\neg_{1}p\right)\right)\in {\tilde{\neg }}_{1}\left(b\right)$$

with:

$${\tilde{\neg }}_{1}\left(b\right)=\left\{\begin{array}{cc} \left[0,1-b\right] & b\in D\\ \left[1-b,1\right] & b\notin D \end{array}\right.$$

This means that the application of the double negation many times not necessarily has as a consequence a concentration of the values of the valuation around 0.

A similar analysis can be made for the second negation presented in this work, and the conclusion—though not identical—goes in the same direction. The principle of double negation is not satisfied either.

### 6.3. Quantum N-Matrix as a Refinement of an F-Expansion of a Finite N-Matrix

In this section, we make use of the following Definition (presented in [72]):

**Definition** **20.** *Let $M_{1}=\langle V_{1},D_{1},O_{1}\rangle$ and $M_{2}=\langle V_{2},D_{2},O_{2}\rangle$ be N-matrices for L.*

*1*. *$M_{1}$ is a refinement of $M_{2}$ if $V_{1}\subseteq V_{2}$, $D_{1}=D_{2}\cap V_{1}$, and ${\tilde{⟡}}_{M_{1}}\left(\bar{x}\right)\subseteq {\tilde{⟡}}_{M_{2}}\left(\bar{x}\right)$ for every n-ary conective ⟡ of L and every tuple $\bar{x}\in V_{1}^{n}$.*
*2*. *Let F be a function that assigns to each x∈V a non-empty set F(x), such that $F\left(x_{1}\right)\cap F\left(x_{2}\right)=\emptyset$ if $x_{1}\neq x_{2}$. The F-expansion of $M_{1}$ is the following N-matrix $M_{1}^{F}=\langle V_{F},D_{F},O_{F}\rangle$, with $V_{F}={⋃}_{x\in V}F\left(x\right)$, $D_{F}={⋃}_{x\in D}F\left(x\right)$, and ${\tilde{⟡}}_{M_{1}^{F}}\left(y_{1},\ldots ,y_{n}\right)={⋃}_{z\in {\tilde{⟡}}_{M_{1}}\left(x_{1},...,x_{n}\right)}F\left(z\right)$ whenever ⟡ is an n-ary connective of L, and $x_{i}\in V$, $y_{i}\in F\left(x_{i}\right)$ for every 1≤i≤n. We say that $M_{2}$ is an expansion of $M_{1}$ if $M_{2}$ is the F-expansion of $M_{1}$ for some function F.*

We now show that the N-matrix constructed for the orthomodular lattice of projection operators for the case of D=1 is a particular refinement of an F-expansion of a finite N-matrix. We will not consider the case with more designated values. In order to reach this aim, we must give first some definitions with regard to expansions and refinements. For a more detailed treatment of the techniques used in this section (and proofs and propositions), we refer the reader to [73].

We say that $M_{2}$ is a simple refinement of $M_{1}$, if it is a refinement (Definition (20)) and it satisfies $V_{1}=V_{2}$. Given a function *F*, let Im(F) and Dom(F) denote the image and domain of *F*, respectively. For every expansion function *F* and y∈⋃Im(F), we denote by $\tilde{F}\left[y\right]$ the unique element x∈Dom(F) such that y∈F(x).

**Definition** **21.** *Let $M_{1}=\langle V_{1},D_{1},O_{1}\rangle$ and $M_{2}=\langle V_{2},D_{2},O_{2}\rangle$ be N-matrices and F an expansion function for $M_{1}$. We say $M_{2}$ is an F-rexpansion of $M_{1}$ if it is a refinement of the F-expansion of $M_{1}$. It is called:*

*1*. *simple if it is a simple refinement of the F-expansion of $M_{1}$.*
*2*. *preserving if $F\left(x\right)\cap V_{2}\neq \emptyset$ for every $x\in V_{1}$.*
*3*. *strongly preserving if it is preserving, and for every $x_{1},\ldots ,x_{n}\in V_{2}$, $⟡\in {⟡}_{L}^{n}$ and $y\in {\tilde{⟡}}_{1}\left(\tilde{F}\left[x_{1}\right],\ldots ,\tilde{F}\left[x_{n}\right]\right)$, it holds that the set $F\left(y\right)\cap {\tilde{⟡}}_{2}\left(x_{1},\ldots ,x_{n}\right)$ is not empty.*

Loosely speaking, being a preserving rexpansion amounts to keeping at least one “copy” of every original truth-value. Being strongly preserving means that this property holds not only for the set of truth-values, but also for the interpretation of the connectives.

**Proposition** **2.** *Every simple rexpansion is preserving, every expansion is a strongly preserving rexpansion, and every preserving rexpansion of a matrix is strongly preserving.*

The proof can be found in [73].

**Proposition** **3.** *The N-matrix $M_{2}=\langle V_{2},D_{2},O_{2}\rangle$ is a rexpansion of the N-matrix $M_{1}=\langle V_{1},D_{1},O_{1}\rangle$ iff there is a function $f:V_{2}\to V_{1}$ such that:*

*1*. *For every $x\in V_{2}$, $x\in D_{2}$ iff $f\left(x\right)\in D_{1}$.*
*2*. *For every $x_{1},\ldots ,x_{n}\in V_{2}$ and $y\in {\tilde{⟡}}_{2}\left(x_{1},\ldots ,x_{n}\right)$, it holds that $f\left(y\right)\in {\tilde{⟡}}_{1}\left(f\left(x_{1}\right),\ldots ,f\left(x_{n}\right)\right)$.*

**Proposition** **4.** *If $M_{2}$ is a rexpansion of $M_{1}$ then ${⊢}_{M_{1}}\subseteq {⊢}_{M_{2}}$. Moroever, if $M_{2}$ is strongly preserving, then ${⊢}_{M_{1}}={⊢}_{M_{2}}$.*

Now we proceed to find a finite N-matrix of which our quantum N-matrix is an expansion. This N-matrix will not be unique, given that there exist infinitely many rexpansions for a given N-matrix, and each matrix can be the rexpansion of different N-matrices. Each one of these rexpansions is compromised with different expansion functions and different degrees of refinement. Once one of these N-matrixes is found, it is possible to use Proposition (4) in order to relate the sets of theorems.

Let $V_{2}=\left[0,1\right]$, $V_{1}=\left\{t,T,F\right\}$, $D_{2}=\left\{1\right\}$ and $f:V_{2}\to V_{1}$, such that

f(1)=t;f(0)=F;f(α)=T,α∈(0,1)

In this case, we aim to find a finite N-matrix of three values, in such a way that the quantum N-matrix be its expansion. We then propose an N-matrix of two values.

Applying item 1 of Proposition (3): x∈{1} iff $f\left(x\right)\in D_{1}$$\Rightarrow D_{1}=\left\{t\right\}$.

Now we apply item 2 of Proposition (3) in order to find the interpretation set for each connective.

$$\forall x_{1},x_{2}\in \left[0,1\right],\;\;y\in {\tilde{\vee }}_{Q}\left(x_{1},x_{2}\right)=\left[\max\left(x_{1},x_{2}\right),1\right]\Rightarrow f\left(y\right)\in {\tilde{\vee }}_{1}\left(f\left(x_{1}\right),f\left(x_{2}\right)\right)$$

If $x_{1}=x_{2}=0$, then $y\in \left[0,1\right]\Rightarrow f\left(y\right)\in {\tilde{\vee }}_{1}\left(f\left(0\right),f\left(0\right)\right)$. Thus,

(27) $${\tilde{\vee }}_{1}\left(F,F\right)=\left\{t,T,F\right\}$$

If $x_{1}=x_{2}=1$,

$$y\in {\tilde{\vee }}_{Q}\left(1,1\right)=\left[\max\left(1,1\right),1\right]=\left\{1\right\}\Rightarrow f\left(1\right)\in {\tilde{\vee }}_{1}\left(t,t\right).$$

Then,

(28) $${\tilde{\vee }}_{1}\left(t,t\right)=\left\{t\right\}$$

If $x_{1}=0,x_{2}=\alpha ;\alpha \in \left(0,1\right)$, $y\in \left[\alpha ,1\right]\Rightarrow f\left(y\right)\in {\tilde{\vee }}_{1}\left(F,T\right)$.

(29) $${\tilde{\vee }}_{1}\left(F,T\right)=\left\{t,T\right\}$$

By following this procedure, it is possible to find all the elements of the interpretation set of the conjunction. The following table resumes all the results of a possible candidate:

(30) $$\begin{array}{cccc} {\tilde{\vee }}_{1} & t & T & F\\ t & \left\{t\right\} & \left\{t\right\} & \left\{t\right\}\\ T & \left\{t\right\} & \left\{t,T\right\} & \left\{t,T\right\}\\ F & \left\{t\right\} & \left\{t,T\right\} & \left\{t,T,F\right\} \end{array}$$

It is important to remark that the interpretation set for the disjunction is one of several possibilities. That in this case, we are not imposing the constrains related to Gleason’s theorem. In the same way, it is possible to find the set corresponding to the conjunction. The results are shown in the following table:

(31) $$\begin{array}{cccc} {\tilde{\wedge }}_{1} & t & T & F\\ t & \left\{t,T,F\right\} & \left\{F,T\right\} & \left\{F\right\}\\ T & \left\{T,F\right\} & \left\{T,F\right\} & \left\{F\right\}\\ F & \left\{F\right\} & \left\{F\right\} & \left\{F\right\} \end{array}$$

For the negation, we have:

(32) $$\begin{array}{cccc} & t & T & F\\ {\tilde{\neg }}_{1} & \left\{F\right\} & \left\{T\right\} & \left\{t\right\} \end{array}$$

For the above table we have taken the standard negation associated to the lattice.

If $V_{1}=\left\{t,F\right\}$ is chosen as the initial set (instead of $V_{1}=\left\{t,T,F\right\}$), it is possible to proceed as follows:

$$f:V_{2}\to V_{1}$$

such that f(1)=t, f(α)=F and α∈[0,1). Proceeding in an analogous way as before, we arrive at the following table:

(33) $$\begin{array}{ccc} {\tilde{\vee }}_{1} & t & F\\ t & \left\{t\right\} & \left\{t\right\}\\ F & \left\{t\right\} & \left\{t,F\right\} \end{array}$$

and a similar procedure can be applied to the rest of the connectives.

## 7. Conclusions

In this work we have seen that:

- There are several ways in which one can affirm that the quantum formalism does not obey truth functionality.
- The set of projection operators admits N-matrices, and thus, the N-matrices formalism can be adapted to quantum mechanics.
- Each quantum state can be interpreted as a valuation associated to a non-deterministic semantics. Indeed, the set of quantum states can be *characterized* as being equivalent to the set of valuations defined by the N-matrices that we propose in Section 5. We have proved that quantum states, considered as valuations, are, in general, dynamic and non-static. We have provided a similar analysis for generalized probabilistic models.
- There exist different candidates for non-deterministic semantics which are compatible with the quantum formalism. We have studied different examples.
- It is possible to give a notion of a logical consequence associated to non-deterministic semantics in the quantum formalism (a study that should be extended, of course, in future work).

We think that the constructions presented here can open the door to interesting questions in both, the fields of quantum mechanics and logic. On the physics side, it opens the door to studying axioms for generalized probabilistic systems using logical axioms. On the logical side, it gives place to a new model of N-matrices with possible physical applications.

## Figures and Tables

**Table 1:** Deterministic vs. non-deterministic matrices.

	Deterministic Matrices	N-Matrices
Truth values set	V	V
Designated values set	D⊂V	D⊂V
Connectives ⟡	$\tilde{⟡}:V^{n}\to V$	$\tilde{⟡}:V^{n}\to P\left(V\right)\setminus \left\{\emptyset \right\}$
Valuations	Non-dynamic	Possibly dynamic and possibly non-static
Truth-Functional	Yes	Not necessarily

**Table 2:** Table comparing the different valuations that can be defined on classical vs. quantum propositional systems.

	Classical systems	Quantum systems
Lattice	Boolean Algebra	Projections lattice
Truth-tables	Admit deterministic matrices	Only proper N-matrices
Truth-Values	Admit valuations in {0,1}	Only valuations in [0,1]
Truth-Functional	Yes (for deterministic states)	No
Satisfy Adequacy	Yes (for deterministic states)	No
